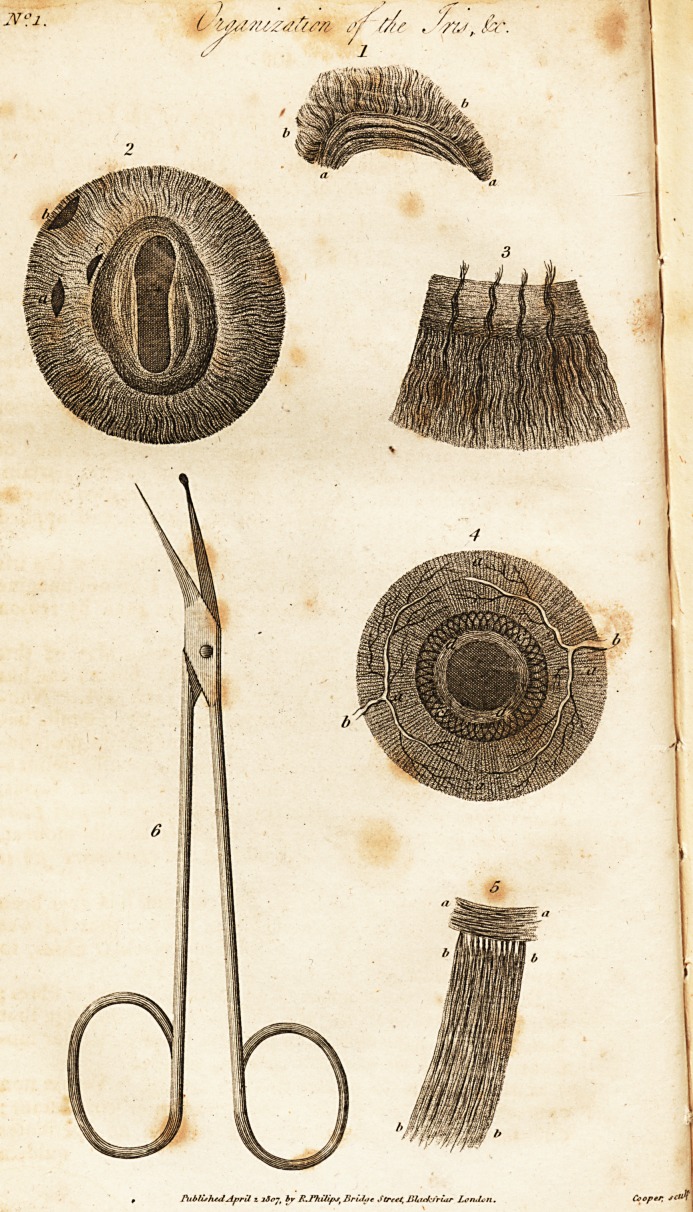# Two Memoirs on the Organization of the Iris, and on Artificial Pupils

**Published:** 1807-05

**Authors:** J. P. Maunoir, Thomas Young

**Affiliations:** Surgeon, of Geneva; late of Coleman Street, now of Finsbury Square, Surgeon


Two Memoirs *on the* Organization *of the* Iris, *and on* artificial Pupils, *by Mr.* J. P. Maunoir, *Surgeon, of Geneva. Translated by Mr.* Thomas Young, *late of Coleman Street, now of Finsbury Square, Surgeon.*

( With Engravings. )

### FIRST MEMOIR.

THE alternate motion of contraction and dilatation of the pupil is still a problem for physiologists.

Porterfield believed that the choroid having arrived at the ciliary ligament, separated, and sent muscular fibres backward to the crystalline, and a double plane of fibres, of the same nature, forwards, of which the posterior lamina spreads itself, in the form of rays, upon the posterior face of the Iris, and the anterior lamina, formed of circular fibres, composed the anterior face of that curtain.

He admitted also, that in the iris there are two muscles of the same size, occupying all its extent, and applied one upon the other.

He added, however, " All anatomists think that the iris is provided with circular fibres, although I do not imagine that one can demonstrate them otherways than by reason and analogy."

Ruysch, Morgagni, Whytt, Hunter, were also of this opinion; all have seen the radiating fibres, but no one has ever demonstrated the orbicular ones. Zinn says, " Nunquam autem, neque in oculis humanis neque in oculis bubulis vidi fibras orbiculares, sive anteriorem sive posteriorem faciem iridis examinaverim, quales Ruyschius delineavit, et descripsit; etsi ille dubius de illis loquatur fassus, *illas fibras orbiculares non tam luculenter conspici posse quin oculi mentis in auxilium sint vocandi;* & alibi moneat, *se tantum circulum minorem præditum esse existimare fibris orbicularibus.*"

Zinn, after having declared, that no one had ever been able to discover the circular fibres, avows, that he was tempted to admit this double order of muscular fibres, to explain, by analogy, the motions of the Iris.

Duverney does not admit, that there are circular fibres; he merely says, that we are obliged to acknowledge that Nature may have means, to perform these particular motions, of which we have, at present, no idea.

Haller has cut the matter short in saying, " Verum non oportet fabricas excogitare quas sensus non confirmant; circulus in uveâ constrictor nullus est, sed ne aperientes quidem quidem fibræ manifestæ, neque in fele, cui accuratissimè pupilla constringitur, de sphinctere cogitari potest." In the mean time, in saying "fabricas non oportet excogitare," his brilliant imagination had created an ingenious system; he had considered each radiating fibre as a cavernous body, which, by the influx or determination of a larger quantity of fluid is put into a state of erection, and thus being elongated they diminish the aperture of the pupil.

Blumenbach thought to explain all by saying, that there does not exist any fleshy fibres in the texture of the Iris, but that its movements depend upon a force or an action *sui generis*. To give such a solution of the Problem, is to avow that it is not to be solved.

Desiring to extricate myself from the doubts into which these divers opinions had thrown me, and hoping by the help of the microscope to discover something new in the structure of the Iris, I began my researches in the following manner: I caused several Irises taken from the human eye to be macerated in water, and after several days, I agitated them, to get rid of their varnish* and render them transparent; I then placed one of these membranes under a strong lens, and I easily discovered its radiating fibres, they appeared to me to be so many little hollow cylinders, having no communication with one another, sometimes running parallel in a winding or serpentine form, sometimes riding on each other, and composing altogether the ciliary ring; † I increased the power of my glasses, but was not able to trace the radiating fibres beyond the little circumference of the ciliary ring; on the contrary, I plainly perceived them abruptly terminating at (without penetrating) the pupilary ring.

As for the fibres of this (viz. the pupilary ring) I never could perceive, or discover, what figure or direction they assumed.

The pupilary ring was formed of so fine and compact a texture, that the strongest magnifier would not assist me to develope its structure, all I could perceive was, that this structure was not the same as that of the ciliary ring. I hoped

* By *varnish* is meant the nigrum pigmentum.

† Almost every Iris is divided by a *trace* or circle, (often of a different colour from the rest of the Iris), which makes two distinct portions of this membrane, and points out the separation of the two muscles; one comprised between this trace and the ciliary ligament forms the *Ciliary Ring*, the other comprehended between this trace and the pupil, forms the *Pupilary Ring*.

I hoped, that the iris of the ox might afford some assistance in this inquiry. In this animal the Iris is of a very thick and compact texture, the pupil is transversely eliptic, and forms in its greatest state of contraction, a transverse line. Its anterior or visible surface, is of a brownish grey, which is of the deepest colour in the PUPILARY RING, its posterior face or uvea is covered with the most beautiful black varnish.* The ciliary ring, on this surface, presents to view, striated or radiated folds, resembling radiated flowers; but the *pupilary ring* offers nothing like this, it is altogether perfectly smooth. (See Cuvier.)

Having perfectly cleared one of these membranes, (the ox's iris) from its varnish or nigrum pigmentum by washing it, and agitating it in water, which iris had been taken from a fresh eye, I placed it under the microscope; its thickness deprived it of all transparency, I could discover nothing of its structure; I tore off several very thin laminæ, in which, I thought I perceived the fibres disposed in parallel and radiating stria; but this view so taken was by no means satisfactory. I have been speaking of portions taken frnm the ciliary ring, those which I afterwards took from the pupilary ring offered the same density, the same *finesse*; in one word, a structure as obscure as that of the human iris. I began to despair of resolving this beautiful problem.

In the mean time, however, I macerated several eyes of oxen in water; at the end of a month they had contracted a disagreeable smell of putrefied flesh; nevertheless, the different parts of the eye had preserved their ordinary appearance, except the retina, which was dissolved. I agitated an iris for a long time in water, to cleanse it from its colour and mucus; by this means it acquired, (notwithstanding it lost none of its thickness) more transparency than the portions which were taken from the fresh eyes.

I placed a segment of it under the microscope. The portion belonging to the ciliary ring yet preserved on its posterior surface, (which respects the chrystalline), its striated and folded disposition; but in many places, or at many points, the stria were effaced, and one could see, in the most distinct manner, the arrangement of the radiating fibres, the assemblage of which I shall call in *future* the *radiating muscle* or *dilator of the pupil.*

These fibres were disposed in parallel bundles; they commenced

---

* The nigrum pigmentum.

menced at the ciliary circumference, and they terminated, almost abruptly, at the larger circumference of the pupilary ring; some of them, however, seemed to penetrate or enter into it.

Here began another order of fibres, upon which, those which I have just described, fell perpendicularly—these followed the circular direction, (or rather eliptic) of the pupilary ring, and existed in its full extent, that is to say, from the termination of the radiating fibres to the free border of the pupil.

These fibres, forming concentric bundles, I shall call The ORBICULAR MUSCLE or SPHINCTER of the IRIS.

If the Iris be formed of two concentric muscles, one external of radiating fibres, and one internal of circular; one might determine a priori, the result of different incisions which may be made upon this membrane.

An incision parallel to the fibres of one or other of these muscles, would close itself spontaneously.

An OBLIQUE incision would leave an opening in the Iris, smaller in proportion as the number of fibres cut through be less considerable.

An incision perpendicular to the fibres of either muscle, will remain open to the full extent of *its* length; this last kind of incision, made in the DILATOR MUSCLE, will have a form relative to the place in which it shall have been made; if it be made in the middle of the fibres of this muscle, their contraction will be uniform, that is to say, the two borders or lips of the incision will retract equally, and leave an opening, as an artificial pupil, of the form of a weaver's shuttle; it would resemble the pupil of the cat.

If the incision be made *near the* EXTREMITY of the *fibres of the dilator muscle*, the contraction will not be uniform in the TWO BORDERS; EACH SIDE will contract itself to the FULL EXTENT OF THAT CONTRACTION WHICH BELONGS TO THE LENGTH OF ITS FIBRES—or, in other words, *of which the fibres are capable.* There will be scarcely any contraction on the side where the fibres are very short, and will be considerable on the side where the fibres are long; so that the opening or pupil, will have the form of the space comprised between the bow and its chord. This incision made near the ciliary ligament, will give a pupil, of which the external or ciliary lip, or border, will be straight, or nearly so, and the pupilary lip or internal border, semicircular.

But, on the contrary, this incision made near the sphincter ter muscle, will have its internal border straight, and its external curved.*

If it were possible to take off an eliptical portion of the radiating (or dilator) muscle of the Iris, of which the large diameter were perpendicular to its fibres, one should have a spherical pupil. After these general hints, it will be easy to conceive what form of opening each kind of incision will produce, in any given part of the Iris.

*Cémoreau*, agriculturer of Janay near Coppet, about two leagues from Geneva, 58 years of age, of a good constitution, and of a tranquil mind, had been blind two years; the left eye had been lost from his infancy, and was merely a membranous tubercle; he had a cataract in his right, with every favourable symptom for the operation. I performed the operation of extraction, with the bistoury of *Wenzel*, the 1st of November, 1801. Nothing particular occurred in the operation; it was speedily done, and left a perfectly round, clear, and black pupil. He saw, and had his eye set at liberty on the fourth day; when it happened that his chamber, from a fire imprudently lighted in it, was filled with smoke; from that instant, his eye became painful, it inflamed, and very soon presented all the characters of the most violent *Chemosis*.

Of all the remedies which were employed to stop this inflammation, a blister applied to the whole of the occipital region, appeared to have most effect.

At last, after three weeks duration, this inflammation ceased, and the result was, a secondary cataract, a diminution of the opening of the pupil, and its entire immobility on a sudden transition from darkness to light; and, vice versa; nevertheless, he could distinguish darkness from light.

I sent him back into the country, desiring him to return after some time, and expect benefit from a new operation. About the end of March, 1802, he came back again to Geneva; at that time, the pupil was almost obliterated, I say almost, because one could see a small white point in the centre of the pupil, which, perhaps, belonged to the opaque capsule of the crystalline.

The eye being in every other respect sound, healthy, and favourable for my design, I determined to make an incision

* It should seem, that it was this kind of incision, which Demours used to make, and by which he restored M. Sauvage to sight.

incision at the superior part of the Iris, above the place where I was led to suppose the secondary cataract was.

For this purpose, the patient was placed upon a bed near a window, so that the light might fall obliquely upon the eye; the eye-lids were held open by means of two fingers; I made an incision, with the cataract knife, in the external border of the transparent cornea, in its superior part: this incision was exactly the beginning of that which I should have made for the purpose of extracting a cataract, and was three lines in length.

There flowed but little aqueous humour; I introduced into this opening, particular scissars constructed for the purpose, of which I shall give a description.

I cut the Iris transveresely between its superior third, and its two inferior thirds, leaving untouched the pupilary ring, and dividing, perpendicularly, the fibres of the ciliary ring, very near their union with the fibres of the former. There was, in the moment of the incision, a small fold of the Iris, which produced a little rag, (or jagged irregularity), at the nasal extremity of the new pupil, which, afterwards, became entirely effaced.

This pupil being pressed against the cornea, in consequence of the evacuation of the aqueous humour, had, at first, an irregular form, but the patient easily distinguished the hands of my watch, and felt no pain. A small bandage of gummed silk, applied over the closed eye-lids to restrain their motion, was the only dressing. On the third day the eye was set at liberty; on the fifth, the patient quitted his bed; and, on the eight day from the operation, I conducted him to the house of our Prefect M. D'Eymar, where Volta had assembled the members of the Society of Physic and Natural History. There (by candle-light) he distinguished a very small seconds hand of a watch, and was examined by more than twenty persons; his eye was entirely exempt, not only from inflammation, but even from the least of that redness, which is almost always the consequence of the operation for the cataract. The pupil has a regular figure, it presents a transverse hole perfectly black, two lines in length, one line in height in the middle; of which, the inferior border is straight, and the superior semicirculær.

SECOND

### SECOND MEMOIR.

Most of the phenomena, which the different classes of animals present to us, are owing to muscular motion, but the principle of this motion is applied in a manner extremely varied, of which, we cannot always give an account.

Since Everard Home has discovered and demonstrated the muscularity of hydatids; since he has described the muscle of the membrane of the tympanum, in the greater part of animals; since we have traced the muscular fibres of the tænia hydatigena of the liver of a mouse; since we can number and describe, as Lyonnet has done, all the muscles of a caterpillar; and, in short, as muscles are easily traced in all insects, large enough to be dissected, on what ground can we refuse to admit their existence in all parts of animals susceptible of spontaneous motion, in the penniform antennæ of the ephemeria, as also in all the animalculæ found in infusions?

This (the muscular structure) is a means of action, so universally distributed through the animal kingdom, that we are, in some sort, justified in concluding that WHERE there is motion dependant upon vitality, THERE is muscular organization.

The muscular fibres are not essentially red, the colour varies in the different classes of animals, and even in the different parts of the same animal.

The character of muscle is, that it is composed of parallel or diverging fibres, susceptible of being shortened and lengthened in the direction of their axis.

Whenever, therefore, we find this fibrous structure connected with a corresponding motion, we are obliged to admit that there is a muscular organ.

It is now eighteen months, Gentlemen, since I had the honour to read a former Memoir on the muscularity of the Iris; after having there noticed the different opinions of physiologists, upon the cause of the motions of this membrane, I gave an account of my own experiments on the Iris of different animals, and I described two muscles, one circular, the other radiated, found in the iris of an ox; I deduced from this structure certain consequences relative to the manner of making an artificial pupil, and the possibility of determining a priori, the form which every possible incision made upon the iris would assume.

The Memoir was terminated by the history of an artificial pupil, made with success upon a man fifty-eight years of age.

Professor Pictet did me the honour to read my work to the National Institute; and M. Sabattier, appointed by the Class of Mathemetical and Physical Sciences, to render them an account of this Memoir, did it in the following Report.

" Sitting of the 2d Thermidor, An. 10. I have been charged by the Class, to give them an account of a Memoir on the Organization of the Iris, and on an artificial Pupil, which had been read to them by Mr. Pictet, and of which Mr. Maunoir is the author. In the first part of this Memoir, after having given an account of what anatomists have said upon the structure of the iris, he gives the result of his own observations upon the subject. His first experiments were unsuccessful, but at length, after a long maceration, and by the help of a microscope, he discovered two orders of fibres in the iris of the ox; of these fibres, the one occupied the large circumference of this membrane, the other the small one, and surrounded the pupil. Agreeably to this disposition of the fibres, Mr. Maunoir determines the form which an artificial pupil must assume, according as the opening shall be made in the middle of the larger circle, or nearer to the internal or external circumference.

" The second part of Mr. Maunoir's Memoir, contains the history of an artificial pupil, which he effected by making an incision in the iris, in the middle of its breadth. The pupil was almost entirely closed after the extraction of a cataract, which had been rendered unsuccessful by circumstances altogether foreign to the operation. All that remained of this opening *(the natural pupil)* had the appearance of an opaque white spot, which might be considered as a secondary cataract.

" The patient could, however, distinguish light from darkness. This circumstance determined Mr. Maunoir to operate. After having cut the cornea with a cataract knife, to the extent of about three lines, he introduced a pair of scissars of a particular form, (which he proposes to describe elsewhere) into the anterior chamber of the eye, opposite the *superior and middle part of the iris,* and HE TOOK AWAY FROM THIS MEMBRANE A PORTION OF WHICH HE DOES NOT GIVE THE DIMENSIONS. No accident happened, and the patient was conducted, at the end of eight days, to the house of the PREFECT, resident at Geneva, where he was examined by M. Volta, and by twenty other persons, perfectly perfectly cured, and discerning the seconds hand of a watch by candle-light.

"The success of this case adds but little to what was already known, on the possibility of establishing an artificial pupil, in cases where the natural pupil is totally closed. Nevertheless, as these examples are rare, I think that the account of the operations made by M. Maunoir, deserves to be preserved. With respect to his observations on the anatomical structure of the iris, they are so contrary to what has been hitherto said on the organization of this membrane, that it appears to me, that the Class, before they adopt them, should wait till they are confirmed by similar observations."

I must here thank the illustrious Reporter for not adopting my Theory on the Organization of the Iris; his conclusion has engaged me to make new researches, and their result has entirely confirmed that, which I did think ought to have been drawn from the former.

I will only permit myself to re-call attention to one PHRASE of the Report, which makes it appear, either I have expressed myself badly in my Memoir, or that some fault of the copier, unknown to me, has slipt into it; a fault which, if it be founded, would render the observation relative to CEMOREAU insignificant, since it would offer nothing new either in the operative process, or in the results of the operation. This is the phrase of the Report, "and he introduced a pair of scissars, of a particular form, into the anterior chamber of the eye, and opposite the superior and middle part of the Iris, and he *took away from* this membrane a portion, of which he does not give the dimensions." But, observe the phrase of my Memoir, such as it ought to be, and such as in fact it is in the original. "I introduced into this opening particular scissars, . . . . . and I cut the iris transversely, between *its superior third and its two inferior thirds,* leaving untouched the pupilary ring, and dividing perpendicularly the fibres of the ciliary ring, very near their union with the fibres of the former. There was, in the moment of the incision, a small fold of the iris, which produced a little inequality at the nasal extremity of the new pupil, which afterwards became entirely effaced."

It seems to me, that there is nothing in this phrase which leads to suppose the *excision* of a portion * of the iris. I there speak of the division of the fibres of the radiated muscle in a line perpendicular TO these fibres; I there also point out a slight fold of the iris, near the extremity

* The Author's expression is LAMBEAU.

tremity of the incision, which obliged me to cut this membrane doubled, instead of cutting it single; the result of which was, a little pointed tongue, a little portion or ragged point, which *was not taken away*; (to have done this it would have been necessary to have cut its base by a second incision) but which had disappeared on the fourth day, no doubt from the contraction of its fibres. The regularity of Cemoreau's pupil was not therefore deranged but in the moment of the operation, and it was formed by *dividing* the fibres of the iris, and not by *taking away any part of it.* I ought then to add here, or repeat in other words, that since the pupil of Cemoreau was, in the moment of the operation, nothing but a simple slit, (which I might compare to a y laid down (≺ ) of which the tail should be very long) and that the instant after it took the form of the space comprised between a bow and its chord, one must necessarily conclude that there was a contraction of the fibres of the iris, and that the superior border underwent a greater degree of contraction than the inferior, inasmuch as the former was curved and the latter remained almost straight.

Now, must it not be granted, that we have had here all the phænomena which might be expected to result from the muscularity of the iris, such as I have described. But let us now see the new proofs which I have acquired of this organization.

The iris of all animals do not present these two rings, distinct one from the other, as may easily be observed in man and in quadrupeds. They are not to be found in the iris of birds, at least not of those which I have examined; nor in those of fish. On the contrary, they are very easily to be perceived, even with the naked eye, in our domestic quadrupeds, as the dog, the cat, the horse, the ox, the sheep, &c. The iris of these animals presents varieties in the form, in the colour, and even in the arrangement of the muscular fibres; but all have this in common, viz. they are all formed of two rings, one external, *always* radiated; one internal or pupilary, *never* radiated; ordinarily formed of concentric circular or elliptic fibres, but sometimes of a structure so compact and fine, that one cannot assign any particular direction to its fibres, or rather, that one cannot discern them even by the help of a microscope after long macerations.

In vain I sought for these two distinct circles in the iris of birds. This membrane, in this class of animals, always appeared to me to be of the same structure throughout; no no trace of a radiated muscle, but some signs of a circular muscle, occupies the iris altogether. And yet we know it is in the eyes of birds that we must seek for the most beautiful and complete structure; that they have the power of adjustment to distances which our telescopes can scarcely reach; that we perceive in them a movement of the iris, which does not belong to any quadruped, inasmuch as it does not seem to depend only on the difference of light, but upon the will, for in the same minute, and under the influence of the same light, we may perceive alternately the pupil of a bird very large and very small; this phenomenon is particularly apparent in the parroquet and in birds of prey.

I have not yet made many experiments on the eyes of fish, and I am unacquainted with the power of their iris. Cuvier says, that they have little or no movement; among the few I have observed, I have not found the two rings of the iris;* but on clearing the varnish away with a brush, I saw that a part of it, which I could not remove, followed a circular direction throughout all the surface of the iris; which should seem to point out an arrangement of fibres analogous to this disposition, and to arise from the circumstance of the varnish, which could not be removed, remaining lodged in their intervals.

But to return to the iris of quadrupeds. The iris of the horse is circular; its pupil irregularly elliptic; is furnished in its circumference with five or six masses or festoons, which seem to float in the aqueous humour, and are only connected with the border of the pupil by a very slender pedicle, *(vide Fig. 1.)* † The colour of this iris is a deep brown, shaded differently in the two rings, and almost black in the festoons. After having macerated the iris of a horse a long time, and brushed away all the varnish, I could observe the arrangement of its fibres with a lens, and even, by strict attention, with the naked eye. We can there distinguish, as easily as in the iris of the ox, the radiated muscle formed in the ciliary ring, and the orbicular muscle formed in the pupilary ring. But we see here a remarkable difference; it is, that the radiated muscle is furnished at certain distances with bundles of fibres, much thicker and more numerous than those which compose

* The translator has lately examined the iris of a seal and distinctly observed the two rings, and the same arrangement of fibres which the author has discovered in the eyes of other animals.

† I have observed these festoons in the iris of the goat, and I suppose they exist in those of many other animals.

pose the rest. Some of these bundles go as far as the orbicular muscle, cutting its fibres at right angles, and continuing their course as far as the border of the pupil; others lose themselves, and ramify in the festoons already noticed, (*vide Fig.* 3.) We find in the iris of the horse an excess of muscular fibres, and of the black varnish that forms the uvea; for these exuberant fibres, which form the skeleton of the festoons, are enveloped in a great quantity of this varnish, and do not appear to have any other *parenchyma*. What may be the use of these festoons, and of the bundles of radiating fibres, which go to make part of the sphincter muscle? Without doubt the radiating bundles of the sphincter are destined to diminish the energy of its circular fibres, or rather to augment and extend upon it the power of the dilator muscle. To view or consider them apart, we might imagine that each festoon is a particular gland or reservoir of the black varnish of the uvea; but when we reflect on the place which they occupy at the border of the pupil, whose size they diminish, one cannot help attributing another office to them; perhaps they absorb a great number of the luminous rays.

But how shall we explain the difference observable in the particular FORM of the pupils of animals, which in other respects have so much general resemblance, as the dog and the fox? Why, in the first, is the pupil circular, whilst in the second it is elliptic, like that of the cat, and does not approach the circular form but when it is enlarged in the dark? Why have all birds a circular pupil, whilst that of quadrupeds and fish presents so much variety of form?

The solution of this question will, perhaps, one day serve to explain the different mode of seeing in different animals.

It is needless to give the description of the beautiful iris of the cat, on which one sees two rather large vessels, branching, opposed one to the other, and of which the branches anastomose in a manner very apparent. There is scarcely any one who has not taken pleasure in exposing a cat to the light of the sun, for the purpose of seeing its iris expand itself, and its pupil diminish itself to a black line. This iris, after the maceration and cleansing it from its varnish, presents a membrane tolerably transparent, a pupil circular, or nearly so; the pupilary and ciliary rings are very visible; the ciliary is like that which we have seen in other animals, composed of radiating fibres, cut at right angles by the branches of these two large vessels sels sels before mentioned, which are distinctly seen after maceration, *(vide Fig.* 4.) The pupilary ring appears composed of circular concentric fibres, but only for a very small space, near the border of the pupil; the greatest part of its extent, towards the radiated muscle, is formed of curved fibres, which run only through a small part of the circle, and which go some on one side, some on the other, so that they cross or intersect each other, forming a kind of net-work, *(vide Fig.* 4.) The radiated muscle has nothing remarkable; it resembles that of other quadrupeds.

The eye of the bear is comparatively very small for the size of the animal, it is not larger than that of a middle sized dog; its iris is of a very thick texture, and of a very dark brown; and I was not able, by a long maceration, to free it from its colouring matter (the varnish) and give it a sufficient degree of transparency, to distinguish the arrangement of the fibres, of its pupilary and ciliary ring.

I have examined the iris of many other animals, but my time has not always permitted me to take drawings, or to collect the notes of my observations; I will only say, that what I have observed upon the iris of quadrupeds has rendered evident to me the existence of two muscles; and if I have not communicated this evidence to the understanding of my auditors, it is because I had them not by my side to make them observe the same things, or that I have badly related that which I saw.

In the mean time, I confess my opinion was at one time wavering, when from the iris of quadrupeds I went to that of birds; its texture was sometimes too delicate for me to follow, and perceive the order of its fibres; sometimes, and more commonly, it presented only an order of circular fibres; not the least vestige of a radiated muscle; and this in an iris, the movements of which are more rapid and more extensive than in the other classes of animals.

On account of this exception, so important to my theory, I was on the point of renouncing it as illusive, and of being brought back to that of Blumenbach, who thought the movement of the iris depended on an action *sui generis*, an action not to be explained by a comparison with any other known phenomenon; but at last, further observations assisted me to resolve this problem, and confirmed me in my opinion.

Lately one of the swans of the ditch of the New-Gate was killed by a fox, and was given to my brother. I examined the eyes, which are not so large in this bird as I should should have supposed by analogy. In dissecting them, I took away entirely the choroids, still attached to the iris, and macerated them. I examined these membranes; and here follows a copy of a Note, made at the moment of the observation.

Iris of the Swan, examined the 7th of June, 1803. In the iris of the swan, whose eye is very small in comparison with the eyes of other birds, we find an order of orbicular concentric fibres, which appears to occupy its whole extent; near the ciliary ligament this organization is less evident, on account of the thickness and opacity of the iris at this part; but *upon* the ciliary ligament, which is very transparent, one may see clearly *radiating fibres,* which lose themselves in the obscure part already spoken of; and they appeared to me to be the continuation of similar fibres, of which all that part of the choroid is formed which lines the interior of the bony circle; so that in these birds one part of the choroid itself seems to be employed to form the dilator muscle of the pupil, and the sphincter occupies the whole of the iris. If this circumstance be found in other birds, it will serve to explain the extent of contraction and dilatation of their pupil, which seems in this class of animals to depend not only on the passage from darkness to light, and vice versa, but also on their will.

Since I made this remark on the eye of the swan, I recollected that I had had in maceration for some months, the iris of the GRAND DUC united to the whole of the choroid, and I found in these membranes the same disposition of which I have just spoken, but so evident, that the arrangement of the radiating fibres of the choroid and the bundles which are found in the ciliary ligament (and which are but a continuation of them) may be seen very well with the naked eye, *(vide Fig. 5.)*

The power to augment or diminish the extent of the pupil is of greater importance to the adjustment of the eye to different distances than is commonly believed; this faculty is not the principal, but it makes the whole complete; it is not then astonishing that the organization of the eye of birds is far superior in this respect to that of the eye of other animals.

In another Memoir I shall endeavour to shew the relations which the rest of the choroid, the ciliary processes, the marsupium in birds, and all the choroid in other animals, have with the adjustment of the eye to different distances. I shall conclude this Memoir by the history of the the artificial pupils which I have made, and by a description of the process which I followed in performing this operation.

There are many indications to be fulfilled, or, in other words, points to be observed, in an operation of this kind; the omission of one only may render it useless. In the first place it should be observed, that the incision in the cornea, through which the instrument employed to divide the iris is to pass, should not be made opposite the place fixed upon to form the pupil; for if it were so, the more the cicatrix, which is the necessary consequence of the incision of the cornea, should be opaque, the fewer rays of light would be transmitted by the pupil obtained. It will therefore be important to determine before hand the place where this incision should be made. I believe that it may be laid down as a general rule to make it (according to the circumstances of the disease) in the most convenient point of the circumference of the cornea, and at about *two millemeters*, (one line) from its union with the sclerotica, as it is done for the operation of the cataract, preferring, as much as possible, the external side, because the handling of the instrument with which the incision of the iris is made, will always be more easy on the side of the temple, than on the side of the nose, the forehead, or the cheek.

The second indication to be fulfilled, is in case of a partial opacity of the cornea, to make the opening of the iris in the place which corresponds with that part of the cornea, which has preserved the greatest degree of transparency, and to make this incision in the very place which had been determined upon beforehand.

The third, and the most important, is to make THIS INCISION OF THE IRIS, as much as possible, in a direction PERPENDICULAR to that of the fibres to be cut; for then only can one hope that the wound made in the iris will not re-unite, and that a simple slit will change itself into (or become) an elliptic opening.

Now let us take a view of the circumstances, which rendering these indications difficult to be fulfilled, have caused this operation to be considered as very precarious.

The incision of the cornea is easy, it is made as in the operation for the cataract, but it is always accompanied by an escape of the aqueous humour; and the iris applying itself against, or coming in contact with the cornea, it becomes then very difficult, or almost impossible to direct at pleasure a bistory between these two membranes, (viz. the cornea and the iris); we cannot employ any of the usual means means to fix the globe of the eye, because all these means pressing more or less upon it, would occasion the escape of the vitreous humor.

In taking the crystalline and vitreous humor, as the bearing point for the edge of the instrument, the iris flies before the bistory; on the contrary, if we carry the edge of the instrument upon the posterior part of the iris, in taking the cornea for the bearing point, (which is extremely difficult,) either we should not succeed in our attempt to cut the iris, or risk cutting the cornea at the same time. The only manner to direct the bistory easily upon the iris, and to cut this membrane neatly, is to make an incision in the cornea previously as large as for the cataract, but then one is exposed to all the accidents of that operation, which depend almost always upon the wound of the cornea not re-uniting. In the mean time, if there were no other method more easy and less dangerous, to make an artificial pupil, I should prefer it to all others; and it was in following this method, that Mr. Jurine made two upon the same individual, both of which perfectly succeeded.

I will next relate the simple method which I have employed with success, and which I think every oculist may put in practice as easily as myself. I begin by making an incision in the cornea, as much as possible on the external side, (whether there be any opacity at that part or not) about the length of six millemeters (three lines) and at the distance of one millemeter from the sclerotica. This incision should have a curvature parallel to the circumference of the cornea, and in general it will not differ from that which should be made in the operation for the cataract, except that it will be much less; I finish the operation with the instrument, which I shall next describe.

This instrument is a pair of crooked scissars with very thin and narrow blades, of fifteen or eighteen millemeters in length (7 or 8 lines) the bend of their border, near the joint, forming an angle of about 140 degrees; the superior blade, that is to say, that which should be between the cornea and the iris, has its extremity formed like a very small olive. The inferior blade, that is to say, that which is to pass through the iris, and be placed between that membrane and the crystalline, ends in a very sharp point, and cutting on its back, for the extent of about two millemeters, (*vide Fig.* 6.)

The manner of using them is as follows: The scissars are to be introduced flat into the incision of the cornea; when when the point is near the part of the iris where the incision ought to begin, they should be turned in such a manner as that the flat side may be perpendicular to the cornea and the iris; they are to be lightly or gently opened, and then pushed sufficiently, so that the inferior blade shall penetrate into the iris the length that the incision ought to have; then the scissars are to be closed neatly, and the iris will by this means be cut.

This operation is extremely simple and easy, and is performed more quickly than words can well describe. The small incision of the cornea has this great advantage over a larger one, that the movement of the eye-lids cannot separate the lips of the wound, and impede their re-union; a distressing circumstance which too often happens after the operation for the cataract, even when best performed. Here not only THIS ACCIDENT cannot take place, on account of the situation and smallness of the incision, but also we have the inexpressible advantage not to need any bandage, and of leaving the eye altogether free from the first moment. By taking care to keep the patient in a dark chamber during two or three days, one might hope that at the end of this time, or of five or six days at furthest, he would be cured, that his eyes would bear the light, and the aqueous humour be entirely regenerated.

Francis Ninet, tyler, at Lausanne, 39 years of age, received in his youth an injury in his face from the discharge of a gun loaded with powder. Both his eyes were very much injured; the left indeed preserved the power of seeing, but the pupil was torn in its superior and internal part, and from that time its extent was more considerable, and its form irregular. The right was extremely diseased, the cornea became opaque precisely in its centre, and the pupil (which if it had remained entire would have been larger than the opaque spot of the cornea) was withdrawn, and had contracted by its inferior border adhesions with the posterior part of the cornea. In looking from above downwards, one could not see more than a little irregular black hole. Ninet did not know that he could perceive objects with his right eye, as he had from the time of the accident only used his left. Unhappily, four or five years ago the sight of this (the left) began to fail, and was at length entirely lost by an opacity of the crystalline.* It was

* The cataract here mentioned had this peculiarity, the enlargement of the pupil exposed to view a part of the margin of the white crystalline; was then for the first time Ninet perceived that the right eye could give a feeble idea of bright objects. On stooping his head, and turning it to one side, he could perceive an object placed very near him; but when he attempted to lay his hand upon it, he always failed. Upon the whole, his sight did not serve him to guide himself, nor for the ordinary purposes of life. Dr. Scholl sent him to me at Geneva, on the 6th of July, 1802. I performed the operation two days after his arrival. Having placed him on a bed, and myself behind his head, I made with my right hand the incision of the cornea, on the side of the external angle of the eye; then I introduced my scissars into this incision. Having approached the little hole, of which I have spoken, I there introduced the pointed blade of the scissars before described, and I cut at one stroke the pupilary and ciliary ring as far as about two millemeters from the circumference of the iris. This cut of the scissars made only a simple slit, which divided perpendicularly the fibres of the pupilary ring, and separated, almost in a parallel direction, those of the ciliary ring.

Having withdrawn my instrument, I was very much surprised to see the slit which I had just made, scarcely perceptible by a small black line, which marked, if one might use the expression, a ray of the iris. I replaced the scissars in the eye, and in the same manner I cut the iris in the direction of a ray next to the former, forming with it a triangle, of which the base, about three millemeters in breadth, nearly reached the external border of the iris, and of which the summit lost itself near the remainder of the pupil of which I have spoken.

Immediately, (and it was a very beautiful phenomenon) the portion comprised between the two branches of the angle, disappeared, rolling itself up like a spring blind; its summit enlarged itself, and we had a pupil, forming instead of a triangle, a regular parallelogram, *(vide Fig. 7.)* This man having received the visit of a friend (who smoked in his chamber) on the day of the operation, had in consequence of this imprudence, an inflammation which lasted some days, but it did not in the least interrupt his cure; he returned on foot to Lausanne, the 15th day after the operation, distinguishing very well common print, and it appeared like an opaque globe floating in its atmosphere; the gap of the iris, situated between the crystalline and the border of the pupil, did not help him to see, apparently because the light was interrupted by the ciliary processes.

and having relinquished his desire to have the operation for the cataract performed upon his left eye, because he found the recovered sight of the right eye was sufficient for the purposes of his ordinary employ.

It appeared to me that the phenomenon which took place in this operation, may be very well explained in this way.

The border of the old pupil had adhesions with the crystalline; my first incision did not destroy these adhesions, and therefore no contraction could take place; the second incision broke these adhesions, and the portion between the incisions being set free, drew itself up by the contraction of the fibres of the dilator muscle; whilst in the lower part, the divided fibres of the sphincter receded, and formed instead of the point of a triangle, which existed before, one of the little sides of a parallelogram.

I showed Francis Ninet to our Society of Medicine; he was there seen by Messrs. Vieusseux, Odier, Jurine, Veillard, Coindet, Delarive, and Dr. Home, of Edinburgh.— I was assisted in the operation by my brother and the younger M. Peschier; the patient is now at Lausanne, enjoying good health, and seeing well.

I think it unnecessary to give another history in detail of an operation for the artificial pupil, performed upon Madame Renner, of Bienne, 60 years of age; I will only say that after a violent inflammation, in consequence of the operation for the cataract, the pupil was entirely contracted, and the cornea was obscure for a great extent, especially on the external side; I performed the operation on the opposite side in the manner already described; the great movement of the eye during the operation, rendered it very difficult, from whence it happened that the pupil had a very irregular form, which, however, did not prevent her getting well very rapidly, so as to be able to read and write; she was seen at the Society of Natural History, held at my house, the day on which I read this Memoir. There were present Messrs. Count Golowfxin, Tourtoulmine, and the celebrated Count Rumford.

---

### Description of the Plates.

Fig. 1. Is a portion of the iris of an ox macerated, as seen through a magnifier.

a a. A portion of the pupilary or sphincter muscle.

b b. A portion of the ciliary or dilator muscle.

Fig. 2. The iris of an ox of the natural size with its two muscles.

A. The figure of the pupil which would be formed by a transverse slit made in the middle of the dilator muscle.

B. The

B. The figure of a pupil made in the ciliary border of the dilator muscle.

C. The figure of a pupil made by a transverse incision in the pupilary border of the dilator muscle.

Fig. 3. A portion of the iris of a horse viewed through a microscope.

Fig. 4. The iris of a cat.

a. The radiated or dilator muscle.

b b. Two large blood vessels which may be seen in the living subject, whose ramifications go to anastomose upon the external surface of the iris.

c c. A portion of the sphincter muscle, the fibres of which cross each other.

d d. A small portion of the sphincter muscle, of which the fibres are circular and concentric.

Fig. 5. A A. A portion of the iris of a bird, called the Grand Duc, entirely composed of circular fibres.

B B B B. A portion of the internal face of the choroid which lines the bony circle, and is formed of fibres all radiating towards the ris.

Fig. 6. The scissars contrived for the operation of the artificial pupil.

Fig. 7. The Case of Ninet.

A A. A cataract of the left eye, in which is seen the superior border of the crystalline, in consequence of a rent in the iris at its superior part.

B B. The right eye, on which an artificial pupil had been made.

*a.* The pupil in form of a parallelogram.

*b.* A white spot in the cornea.

*c.* The cicatrix of the wound made in the cornea.

Fig. 8. The artificial pupil of Cémoreau mentioned in page 412 of the Second Memoir.

## Figures and Tables

**[ill]o. 2. f1:**
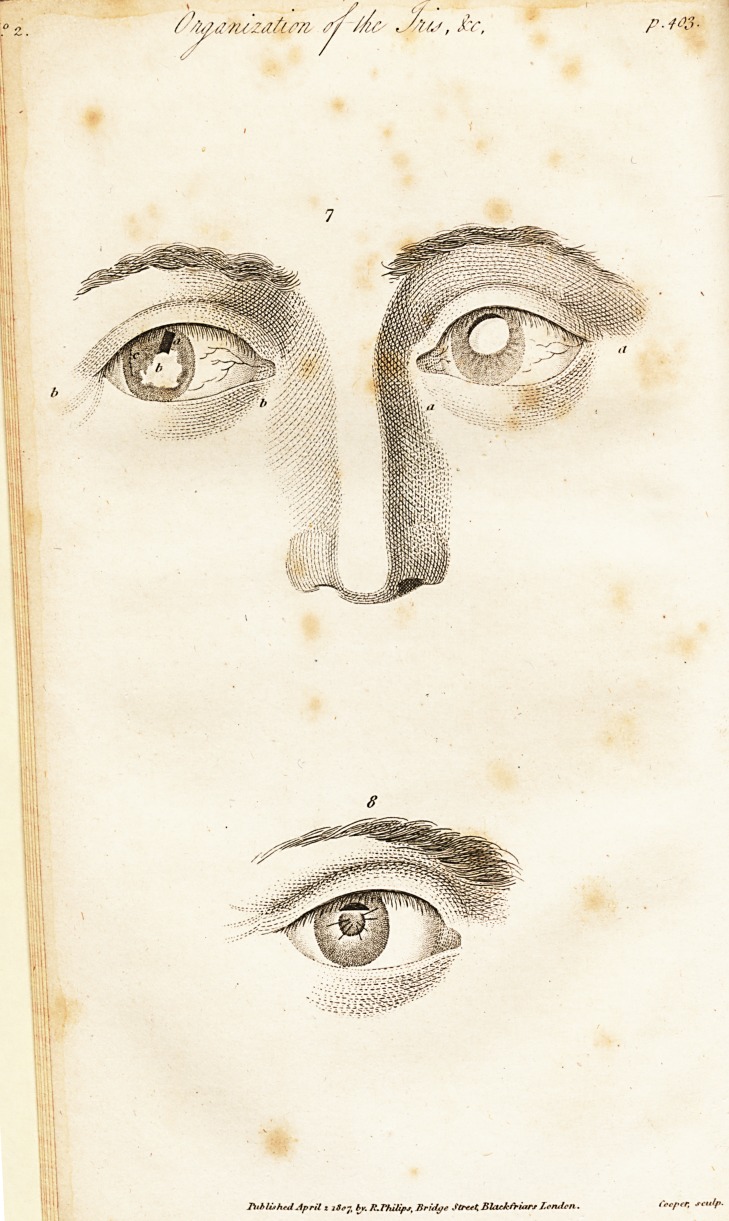


**No. 1. f2:**